# Folic acid ameliorates alcohol-induced liver injury *via* gut–liver axis homeostasis

**DOI:** 10.3389/fnut.2022.989311

**Published:** 2022-10-20

**Authors:** Huaqi Zhang, Yuwei Zuo, Huichao Zhao, Hui Zhao, Yutong Wang, Xinyu Zhang, Jiacheng Zhang, Peng Wang, Lirui Sun, Huizhen Zhang, Hui Liang

**Affiliations:** ^1^Department of Nutrition and Food Hygiene, School of Public Health, Qingdao University, Qingdao, China; ^2^Qingdao Institute for Food and Drug Control, Qingdao, China

**Keywords:** alcohol-induced liver injury, folic acid, gut–liver axis, inflammation, gut microbiota

## Abstract

The gut–liver axis (GLA) plays an important role in the development of alcohol-induced liver injury. Alcohol consumption is typically associated with folic acid deficiency. However, no clear evidence has confirmed the effect of folic acid supplementation on alcohol-induced liver injury *via* GLA homeostasis. In this study, male C57BL/6J mice were given 56% (v/v) ethanol and 5.0 mg/kg folic acid daily by gavage for 10 weeks to investigate potential protective mechanisms of folic acid in alcohol-induced liver injury *via* GLA homeostasis. Histopathological and biochemical analyses showed that folic acid improved lipid deposition and inflammation in the liver caused by alcohol consumption and decreased the level of ALT, AST, TG, and LPS in serum. Folic acid inhibited the expression of the TLR4 signaling pathway and its downstream inflammatory mediators in the liver and upregulated the expression of ZO-1, claudin 1, and occludin in the intestine. But compared with the CON group, folic acid did not completely eliminate alcohol-induced intestine and liver injury. Furthermore, folic acid regulated alcohol-induced alterations in gut microbiota. In alcohol-exposed mice, the relative abundance of *Bacteroidota* was significantly increased, and the relative abundance of *unclassified_Lachnospiraceae* was significantly decreased. Folic acid supplementation significantly increased the relative abundance of *Verrucomicrobia*, *Lachnospiraceae_NK4A136_group* and *Akkermansia*, and decreased the relative abundance of *Proteobacteria*. The results of Spearman’s correlation analysis showed that serum parameters and hepatic inflammatory cytokines were significantly correlated with several bacteria, mainly including *Bacteroidota*, *Firmicutes*, and *unclassified_Lachnospiraceae*. In conclusion, folic acid could ameliorate alcohol-induced liver injury in mice *via* GLA homeostasis to some extent, providing a new idea and method for prevention of alcohol-induced liver injury.

## Introduction

Chronic alcohol ingestion can lead to more than 200 diseases, of which alcoholic liver disease (ALD) is one of the most common and serious diseases (1). ALD is a spectrum of disease ranging from asymptomatic liver steatosis to the development of alcoholic hepatitis, fibrosis, and cirrhosis (2), threatening to the health and lives of millions of people around the world every year.

The gut–liver axis (GLA) refers to the interaction between the gut and liver, which is one of the main pathways for the development and progression of ALD (3). Lipopolysaccharide (LPS), also known as endotoxin, is a key trigger of liver inflammation, which could lead to liver steatosis and inflammatory injury (4, 5). Both alcohol-mediated gut microbial dysbiosis and intestinal barrier destruction enhance the release of a large amount of LPS from the intestine into serum, which is later transported to the liver (4). Leaky LPS can be bound by toll-like receptor 4 (TLR4) (6) on the surface of liver cells, activating the downstream nuclear factor kappa-B (NF-κB) inflammatory pathway, promoting an inflammatory cascade response, inducing an overproduction of inflammatory cytokines (7), and consequently leading to inflammatory injury of the liver. Preclinical and clinical studies have shown that alcohol consumption could affect the amount and composition of gut microbiota (8), leading to dysbiosis of gut microbiota. Dysbiosis of gut microbiota could also affect intestinal barrier function and further damage the liver through microbial products such as LPS. Modulation of gut microbiota is also considered a strategy for the amelioration of ALD, aimed at preventing or delaying liver injury (9).

Folic acid, also known as vitamin B_9_, is a water-soluble vitamin. It is an essential nutrient and a micronutrient necessary for normal human growth and development (10). Notably, folic acid deficiency was found to be one of the most common phenomena of malnutrition in patients with alcoholism. In the United States, a study found that 80% of chronic alcoholics had low serum folic acid levels, and among these chronic alcoholics with low serum folic acid levels, 44% of them were in the severe deficiency range (11). Another study found that 40% of anemic alcoholics had low red blood cell folic acid levels (12). Previous studies pointed out that folic acid could prevent and improve non-alcoholic fatty liver disease (NAFLD) induced by exerting antioxidant and anti-inflammatory effects (13–15), and it has also been found that folic acid had potential to regulate gut microbiota (16). A few studies showed that folic acid could improve alcohol-induced liver injury by exerting antioxidant (17, 18). However, the effect of folic acid on alcohol-induced liver injury *via* GLA homeostasis has not been reported yet.

In this study, alcohol-exposed C57BL/6J mice were given 5.0 mg/kg folic acid for 10 weeks to investigate the effect of folic acid on alcohol-induced liver injury and its mechanism *via* GLA homeostasis.

## Materials and methods

### Animals and experimental design

A total of 24 male C57BL/6J mice (20 ± 2 g, 7 weeks old) were purchased from Beijing Vital River Laboratory Animal Technology Co., Ltd. (License number: SCXK [Jing], 2016-0006). The animals were housed in a specific pathogen-free and environmentally controlled room with constant temperature (22–25°C), humidity (50–60%), and a 12-h light/dark cycle. Animal experiments were performed according to the guidelines of the institutional animal ethics committee and were approved by the Qingdao University Laboratory Animal Welfare Ethics Committee (approval number: 20201030C572720210108044).

**FIGURE 1:**
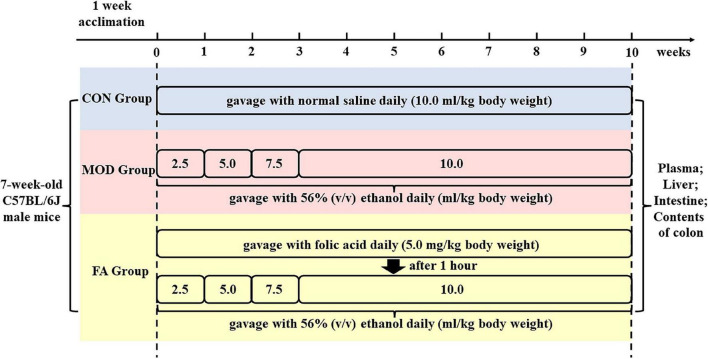
Schedule of the experimental protocol and drug administration of folic acid and ethanol.

After 1-week acclimation period, the mice were randomly divided into three groups (eight mice per group, with no significant difference in body weight among the groups). The control group (CON) was given normal saline at 10.0 ml/kg (body weight) by gavage. The model group (MOD) was given 56% (v/v) ethanol by gavage. The folic acid intervention group (FA) was given 5.0 mg/kg folic acid (body weight) and 56% (v/v) ethanol daily. Gavage was administered daily in all the groups. All the ethanol administration groups were given 56% (v/v) ethanol at 2.5, 5.0, and 7.5 ml/kg (body weight) for the first 3 weeks to acclimatize to the stimulation of ethanol, and then given 56% (v/v) ethanol at 10.0 ml/kg (body weight) for the remaining 7 weeks. The FA group was given ethanol after folic acid intervention for 1 h (Figure 1). Folic acid (≥97% purity, molecular weight 441.40 g/mol) was obtained from Sigma-Aldrich (MO, USA).

At 12 h after the final gavage, the mice were killed, and blood samples were collected from the retro-orbital venous plexus. The blood samples were centrifuged at 3,000 *g* for 10 min at 4°C to obtain serum. The livers were immediately removed and weighed to calculate the liver index (liver index% = liver weight (g)/body weight (g) × 100%). The contents of the colon were harvested into sterile tubes to analyze the structure and composition of gut microbiota. All the samples were stored at -80°C for subsequent experiments.

### Serum biochemical analysis

The levels of serum alanine aminotransferase (ALT), aspartate aminotransferase (AST), triglyceride (TG), and total cholesterol (TC) were measured using an automatic biochemical analyzer (Hitachi High-Technologies Corporation, Tokyo, Japan). The concentration of LPS in serum was measured with the Endpoint Chromogenic Endotoxin Detection LAL Kit.

### Histopathological examination

Liver and ileum histopathology were assessed *via* H&E staining. The samples were fixed with 4% paraformaldehyde. After 24 h, the samples were embedded in paraffin wax. Subsequently, paraffin sections of 5 μm thickness were cut using a sliding microtome (Sakura TTM-200-NO), deparaffinized in xylene, rehydrated in an alcohol gradient, and stained with H&E. Finally, the stained sections were observed with a light microscope (Olympus BX60, Japan). The inflammation histopathological scores of liver tissues were obtained according to a semi-quantitative method, as described in a previous study (19). The average villus height, villus width, and crypt depth of ileum tissue were approximated by measuring these parameters in at least 10 well-oriented villi and crypts per section (20, 21). All measures were carried out by ImageJ software (National Institutes of Health).

### Determination of hepatic inflammatory cytokines

Liver tissue was homogenized in 10% (w/v) phosphate buffer and centrifuged at 3,500 rpm at 4°C for 15 minutes. The supernatant was collected for analysis. The levels of IL-1β, IL-6, and TNF-α in liver tissue were measured using ELISA kits (Nanjing Jiancheng Bioengineering Institute, Nanjing, Jiangsu, China) in accordance with the protocol provided by the manufacturer.

### Western blot analysis

According to our previous methods (22, 23), the total proteins in liver and ileum tissues were extracted, and proteins were quantified using a bicinchoninic acid (BCA) Protein Assay Kit (Nanjing Jiancheng Bioengineering Institute, Nanjing, China). The same amounts of proteins were dissolved on polyacrylamide gels (8–10%) and transferred onto PVDF membranes (Millipore, Bedford, MA, USA). The membranes were blocked with 10% non-fat milk in Tris-buffered saline/Tween (TBST) and then incubated with primary antibodies against TLR4, MyD88, IRAK1, TRAF6 (Proteintech Group, Chicago, USA), IκBα, phospho-IκBα (Santa Cruz, CA, USA), NF-κB, ZO-1, claudin 1, occludin, and β-actin (Cell Signaling Technology, Danvers, MA, USA) at 4°C overnight. After washing with TBST, the membranes were incubated with the corresponding secondary antibodies (Bioeasy, Beijing, China) for 2 h at 37°C. In the end, the bands of proteins were visualized by the Odyssey Infrared Imaging System (Li-Cor Biosciences, Lincoln, NE, USA). β-Actin served as an internal control.

### DNA extraction and 16S rRNA gene sequencing

After 10-week intervention, 16S rRNA gene sequencing was performed on contents of colon samples for microbiome analysis from the CON group, MOD group, and FA group. A total of five samples were randomly selected from each group for sequencing. The sequencing methods were adopted from our previous studies (24, 25). In detail, DNA was extracted using a DNA kit (Tiangen Biotech (Beijing) Co., Ltd.) according to the manufacturer’s instructions. The DNA concentration was measured using a Qubit dsDNA HS Assay Kit and a Qubit 4.0 Fluorometer (Invitrogen, Thermo Fisher Scientific, Oregon, USA). The 338F: 5′-ACTCCTACGGGAGGCAGCA-3′ and 806R: 5′-GGACTACHVGGGTWTCTAAT-3′ universal primer set was used to amplify the V3-V4 region. The total PCR amplicons were purified using Agencourt AMPure XP Beads (Beckman Coulter, Indianapolis, IN) and quantified using the Qubit dsDNA HS Assay Kit and Qubit 4.0 Fluorometer (Invitrogen, Thermo Fisher Scientific, Oregon, USA). After the individual quantification step, amplicons were pooled in equal amounts. For the constructed library, Illumina Novaseq 6000 System (Illumina, Santiago CA, USA) was used for sequencing. According to quality of single nucleotides, raw data were primarily filtered by Trimmomatic. Identification and removal of primer sequences were processed by Cutadapt. PE reads obtained from previous steps were assembled by USEARCH, followed by chimera removal using UCHIME. High-quality reads generated from the aforementioned steps were used in the following analysis. The DADA2 method in QIIME2 was used for the denoise processing after the quality control of data. A total of 0.005% of the number of all sequences sequenced was used as the filtering threshold to filter ASVs. Alpha diversity was calculated and displayed by QIIME2 and R software, respectively. Beta diversity was determined to evaluate the degree of similarity of microbial communities from different samples using QIIME. Principal coordinate analysis (PCoA), analysis of similarities (ANOSIM), heatmaps, and unweighted pair-group method with arithmetic mean (UPGMA) were used to analyze the beta diversity. Principal coordinate analysis (PCoA) was performed by using the Bray–Curtis method. Furthermore, we employed linear discriminant analysis (LDA) effect size (LEfSe) to test the significant taxonomic difference among the groups. A logarithmic LDA score of 4.0 was set as the threshold for discriminative features. The raw data of 16S rRNA gene libraries generated during this study are publicly available at the Sequence Read Archive portal of the NCBI^1^ under accession number PRJNA874577.

### Statistical analysis

Statistical analysis was carried out by SPSS 22.0 statistical software (SPSS, Chicago, IL, USA). The experimental data were presented as mean ± standard deviation (SD), and the comparison of multiple groups was statistically analyzed by one-way analysis of variance (ANOVA). When one-way ANOVA gives a significant result, Fisher’s LSD test was used for comparing the difference between two groups. In this study, *p* < 0.05 was considered statistically significant.

For gut microbiota analysis, the Kruskal–Wallis rank test was used, and *p*-values were adjusted for multiple comparisons using the false discovery rate. The correlations between gut microbiota and the indexes related to alcohol-induced liver injury (including serum parameters and hepatic inflammatory cytokines) were determined by using Spearman’s correlation analysis and were corrected for multiple hypothesis testing; ^+^*p* < 0.05; *p < 0.01.

## Results

### Effects of folic acid on body weight and liver index

**FIGURE 2:**
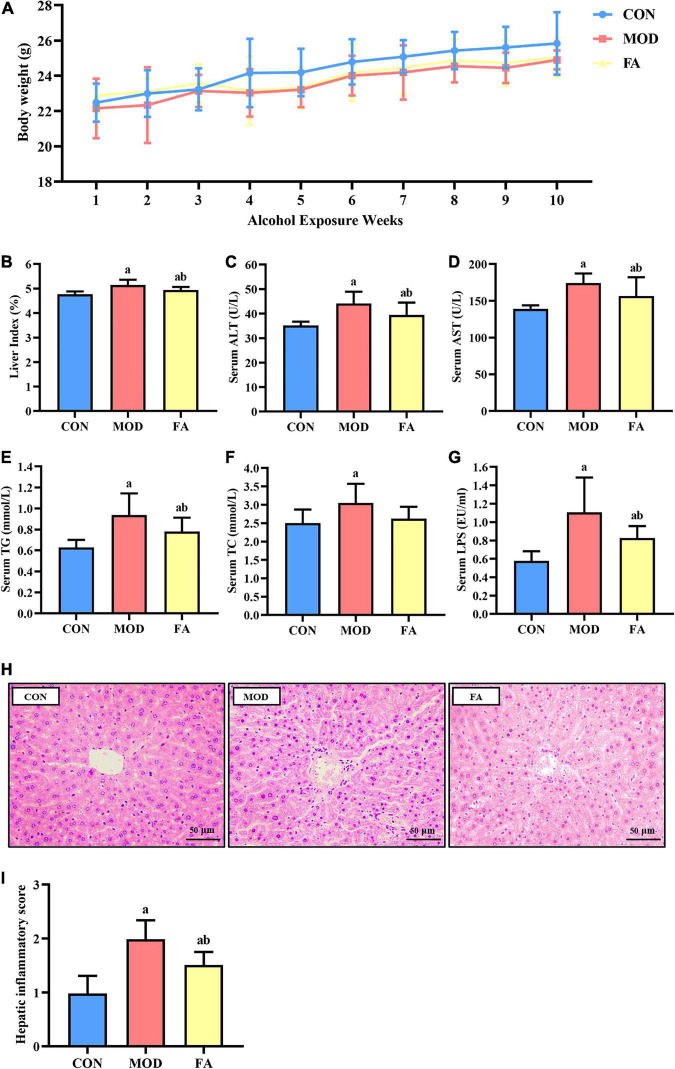
Effects of folic acid on general parameters of mice. **(A)** Body weight; **(B)** liver index; **(C)** serum ALT; **(D)** serum AST; **(E)** serum TG; **(F)** serum TC; **(G)** serum LPS; **(H)** liver histopathology assessed by H&E staining (400×) (*n* = 3/group); **(I)** hepatic inflammatory score. Data are presented as mean ± SD (*n* = 8/group). ^a^*p* < 0.05 compared with the CON group; ^b^*p* < 0.05 compared with the MOD group.

No obvious significant difference in body weight was found among all the groups (*p* > 0.05; Figure 2A). As shown in Figure 2B, the liver index was significantly higher in the MOD group than in the CON group (*p* < 0.05), and it was significantly lower in the FA group than in the MOD group (*p* < 0.05). But the liver index in the FA group was still significantly higher than in the CON group (*p* < 0.05).

### Effects of folic acid on serum parameters

The levels of ALT, AST, TG, and TC were significantly higher in the MOD group than in the CON group (*p* < 0.05). Compared with the MOD group, the levels of ALT, AST, and TG were significantly lower in the FA group (*p* < 0.05), but these parameters in the FA group were still significantly higher than those in the CON group (*p* < 0.05; Figures 2C–F).

The serum LPS level in the MOD group was significantly higher than that in the CON group (*p* < 0.05), and the serum level in the FA group was significantly lower than in the MOD group (*p* < 0.05). But the serum LPS level in the FA group was still significantly higher than that in the CON group (*p* < 0.05; Figure 2G).

### Histopathological examination

#### Effects of folic acid on liver histopathology

In the CON group, the liver lobule structure was clear and complete, and hepatocytes exhibited an ordered arrangement with no evident lipid droplet aggregation and inflammatory cell infiltration. In the MOD group, the hepatic cords of the mice were disordered, and a large number of lipid droplets gathered, accompanied by inflammatory cell infiltration. Compared with the MOD group, liver cords of mice in the FA group were arranged more orderly, and the phenomenon of lipid droplet aggregation and inflammatory cell infiltration improved, but there was still a great difference compared with the CON group (Figure 2H). The results of showed that the hepatic inflammatory score in the MOD group was significantly higher than that in the CON group (*p* < 0.05), and the score in the FA group was significantly lower than that in the MOD group (*p* < 0.05) but was still significantly higher than the CON group (*p* < 0.05; Figure 2I).

#### Effects of folic acid on ileum histopathology

**FIGURE 3:**
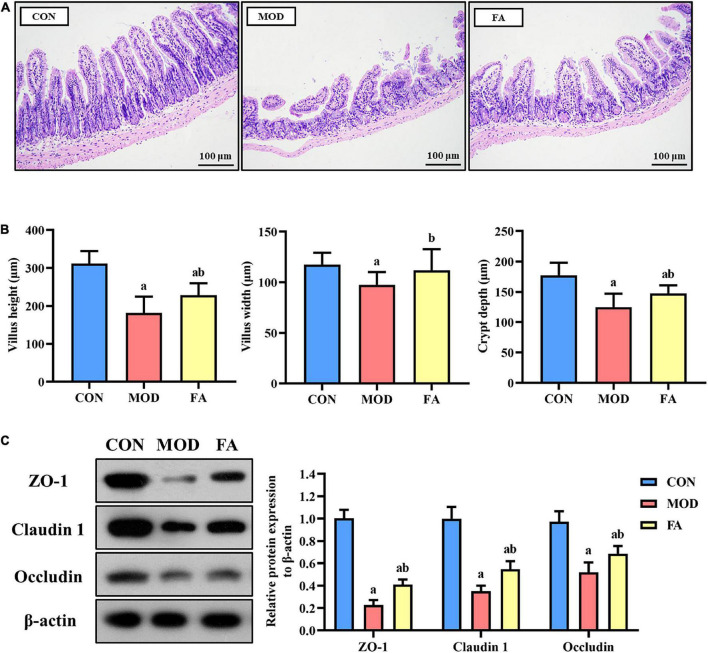
Effects of folic acid on the ileum and the expression levels of tight junction protein. **(A)** Ileum histopathology assessed by H&E staining (200×); **(B)** measurement of villus height, height width, and crypt depth; **(C)** representative Western blot of ZO-1, claudin 1, and occludin protein levels relative to the expression of β-actin in the ileum. Data are presented as mean ± SD (*n* = 3/group). ^a^*p* < 0.05 compared with the CON group; ^b^*p* < 0.05 compared with the MOD group.

The ileum in the CON group was intact in structure and normal in shape. Compared with the CON group, the intestinal villi in the MOD group were disordered, broken, and shortened, the submucosa and muscularis were partially detached. Compared with the MOD group, the intestinal villi of mice in the FA group were arranged more neatly, and the villus height, fracture, and abscission of the lower layer were improved, but there was still a great difference compared with the CON group (Figure 3A). The results of villus measurement showed that the villus height, villus width, and crypt depth in the MOD group were significantly lower than those in the CON group (*p* < 0.05); the villus height, villus width, and crypt depth in the FA group were significantly higher than those in the MOD group (*p* < 0.05), but the villus height and crypt depth were still significantly lower than those in the CON group (*p* < 0.05; Figure 3B).

### Effects of folic acid on the expression levels of tight junction protein in the ileum

The expression levels in the MOD group of ZO-1, claudin 1, and occludin were significantly lower than those in the CON group (*p* < 0.05). However, the expression levels of ZO-1, claudin 1, and occludin in the FA group were significantly higher than in those in the MOD group (*p* < 0.05). But the expression of these proteins in the FA group was still lower than that in the CON group (*p* < 0.05; Figure 3C).

### Effects of folic acid on hepatic inflammatory cytokines

**FIGURE 4:**
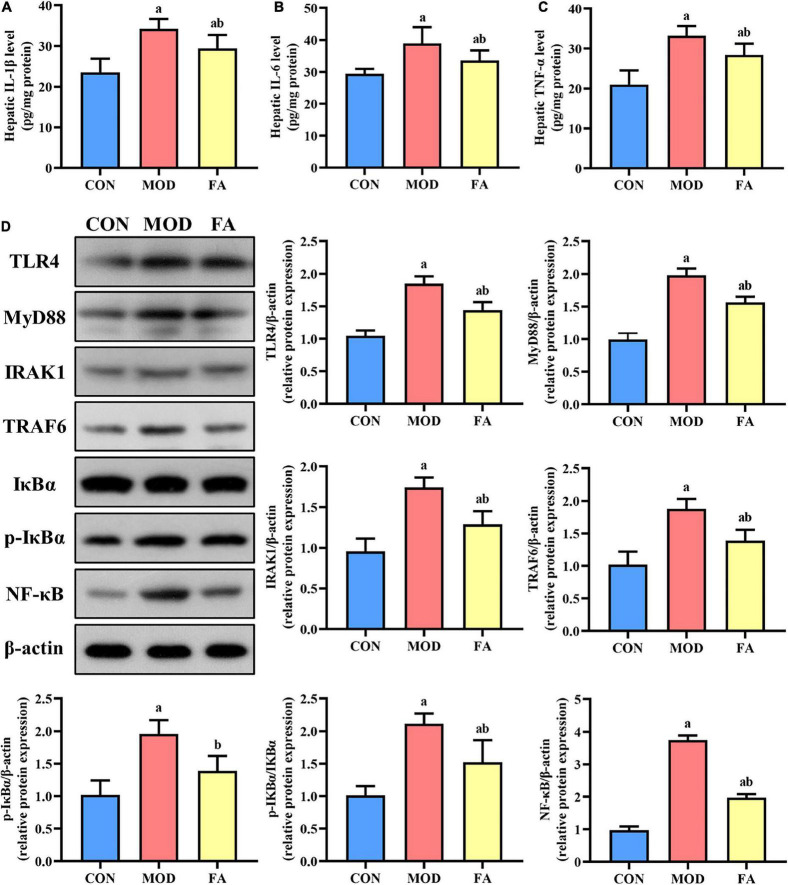
Effects of folic acid on hepatic inflammatory cytokines and the expression levels of TLR4 signaling pathway proteins. **(A)** Hepatic IL-1β level; **(B)** hepatic IL-6 level; **(C)** hepatic TNF-α level; **(D)** representative Western blot of TLR4, MyD88, IRAK1, TRAF6, IκBα, p-IκBα, and NF-κB protein levels relative to the expression of β-actin (*n* = 3/group). Data are presented as mean ± SD (*n* = 8/group). ^a^*p* < 0.05 compared with the CON group; ^b^*p* < 0.05 compared with the MOD group.

The levels of IL-1β, IL-6, and TNF-α in the MOD group were significantly higher than those in the CON group (*p* < 0.05). In the FA group, the levels of IL-1β, IL-6, and TNF-α were significantly lower than those in the MOD group, but these inflammatory cytokines were still higher than those in the CON group (*p* < 0.05; Figures 4A–C).

### Effects of folic acid on TLR4/NF-κB signaling pathway in the liver

The expression levels of TLR4, MyD88, IRAK1, TRAF6, p-IκBα, p-IκBα/IκBα, and NF-κB in the MOD group were significantly higher than those in the CON group (*p* < 0.05), and the expression levels of these proteins in the FA group were significantly lower than those in the MOD group (*p* < 0.05). But the expression levels of TLR4, MyD88, IRAK1, TRAF6, p-IκBα/IκBα, and NF-κB in the FA group were still higher than those in the CON group (*p* < 0.05; Figure 4D).

### Effects of folic acid on gut microbiota

Long-term alcohol consumption could lead to the dysbiosis of gut microbiota. To investigate the effects of folic acid on gut microbiota in alcohol-exposed mice, 16S rRNA gene sequencing was used to analyze gut microbiota of colon contents, and the results are given in the following text.

#### Alpha diversity analysis

**FIGURE 5:**
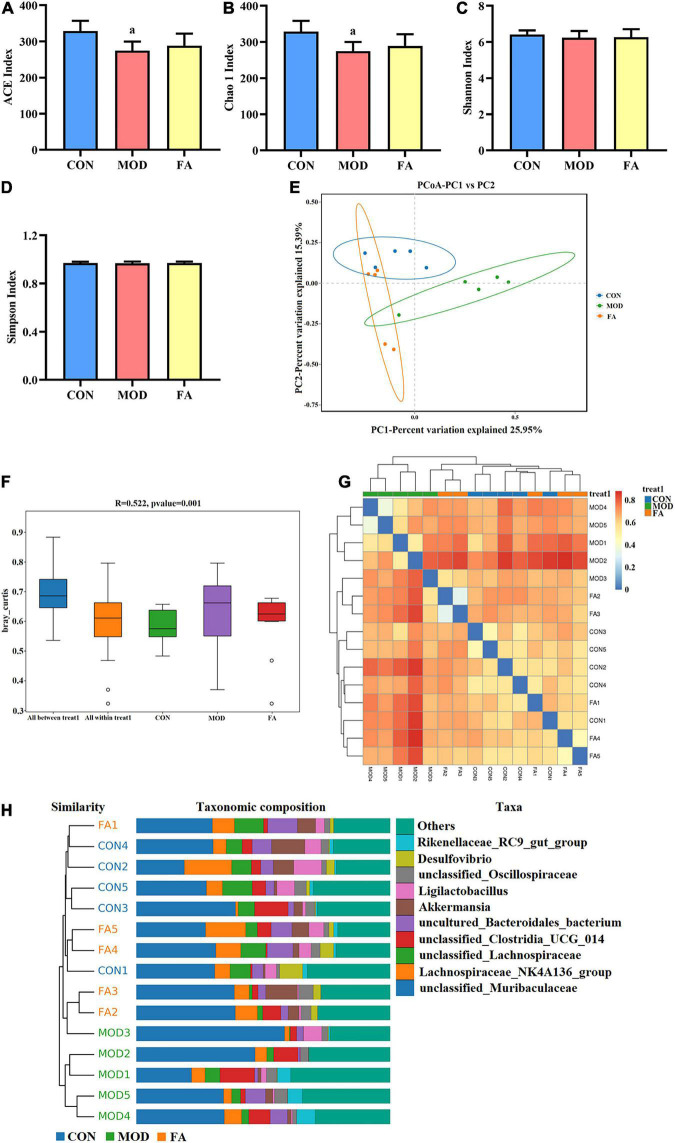
Effects of folic acid on alpha diversity and beta diversity of gut microbiota. **(A)** ACE index; **(B)** Chao 1 index; **(C)** Shannon index; **(D)** Simpson index; **(E)** principal coordinate analysis (PCoA); **(F)** ANOSIM analysis; **(G)** heatmap; **(H)** unweighted pair-group method with arithmetic mean (UPGMA). Data are presented as mean ± SD (*n* = 5/group). ^a^*p* < 0.05 compared with the CON group.

Good’s coverage index of all the groups based on ASV richness was greater than 99.99%. The index of community richness mainly includes ACE index and Chao 1 index, and the results showed that community richness in the MOD group was significantly lower than that in the CON group (*p* < 0.05; Figures 5A,B). The index of community diversity mainly includes Shannon index and Simpson index, and the results showed no statistical significance in community diversity among all the groups (*p* > 0.05; Figures 5C,D).

#### Beta diversity analysis

The principal coordinate analysis (PCoA) showed that the clusters of gut microbiota in the CON group were clearly separated from those in the MOD group, whereas the clusters in the FA group were closer to those in the CON group (Figure 5E). The analysis of similarities (ANOSIM) showed that the difference among the groups was greater than that within the groups (*r* = 0.522, *p* = 0.001; Figure 5F). The heatmap analysis and the unweighted pair-group method with arithmetic mean (UPGMA) showed that the similarity between the FA group and CON group was greater than that between the MOD group and CON group (Figures 5G,H).

#### Relative abundance analysis at phylum level

**FIGURE 6:**
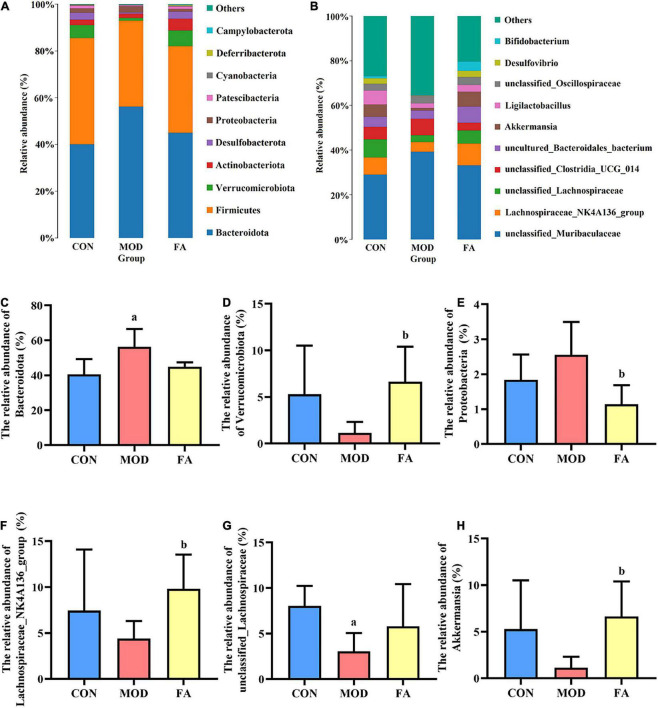
Effects of folic acid on gut microbiota composition at phylum and genus levels. **(A,B)** Composition of gut microbiota at phylum and genus levels; **(C)** relative abundance of *Bacteroidota*; **(D)** relative abundance of *Verrucomicrobiota*; **(E)** relative abundance of *Proteobacteria*; **(F)** relative abundance of *Lachnospiraceae_NK4A136_group*; **(G)** relative abundance of *unclassified_Lachnospiraceae*; **(H)** relative abundance of *Akkermansia*. Data are presented as mean ± SD (*n* = 5/group). ^a^*p* < 0.05 compared with the CON group; ^b^*p* < 0.05 compared with the MOD group.

At the phylum level, gut microbiota mainly composed of *Bacteroidota*, *Firmicutes*, *Verrucomicrobiota*, *Actinobacteriota*, and *Desulfobacterota* (Figure 6A). The relative abundance of *Bacteroidota* in the MOD group was significantly higher than that in the CON group (*p* < 0.05; Figure 6C). The relative abundance of *Verrucomicrobiota* and *Proteobacteria* in the FA group was significantly higher than that in the MOD group (*p* < 0.05; Figures 6D,E).

#### Relative abundance analysis at genus level

At the genus level, top 10 genera from the different groups are shown in Figure 6B. The relative abundance of *Lachnospiraceae_NK4A136_group* and *Akkermansia* in the FA group was significantly higher than that in the MOD group (*p* < 0.05; Figures 6F,H), and the relative abundance of *unclassified_Lachnospiraceae* in the MOD group was significantly lower than that in the CON group (*p* < 0.05; Figure 6G).

#### LEfSe analysis

**FIGURE 7:**
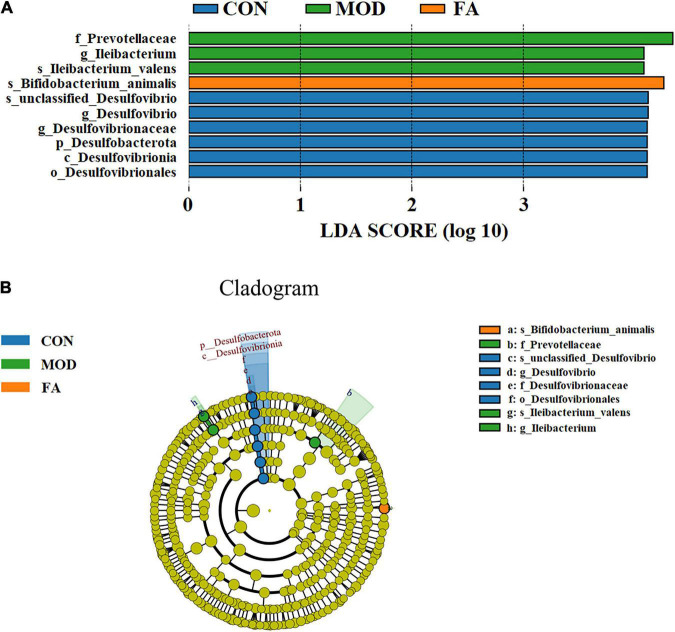
LEfSe analysis. **(A)** Histogram of LDA score distribution (LDA score > 4.0); **(B)** LDA effect size of key bacteria of gut microbiota (*n* = 5/group).

LDA effect size (LEfSe) analysis could be used for comparison among the groups to identify the species with significant differences. LEfSe analysis revealed that 10 ASVs at the phylum (1 ASV), class (1 ASV), order (1 ASV), family (1 ASV), genus (3 ASVs), and species (3 ASVs) showed significant differences among the groups. Among the significantly different ASVs, *p_Desulfobacterota* was the most abundant bacterium in the CON group; *f_Prevotellaceae*, *g_Ileibacterium*, and *s_Ileibacterium_valens* were the most abundant bacteria in the MOD group; and *s_Bifidobacterium_animalis* was the most abundant bacterium in the FA group (Figure 7).

### Statistical Spearman’s correlations between gut microbiota and indexes related to liver injury

**FIGURE 8:**
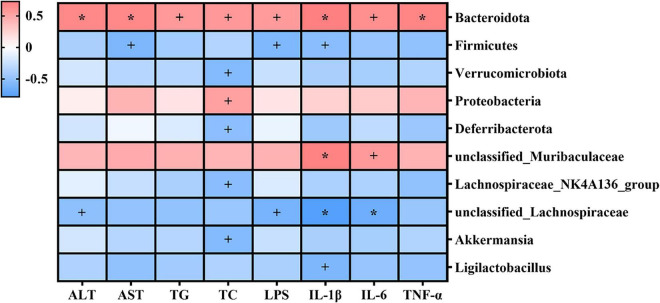
Heatmap of Spearman’s correlations between gut microbiota and indexes related to alcohol-induced liver injury (including serum parameters and hepatic inflammatory cytokines). Blue denotes negative correlation, and red represents positive correlation. ^+^*p* < 0.05; *p < 0.01.

The potential relationships between gut microbiota and the indexes related to alcohol-induced liver injury (including serum parameters and hepatic inflammatory cytokines) were explored by Spearman’s correlation analysis. As shown in Figure 8, *Bacteroidota*, *Proteobacteria*, and *unclassified_Muribaculaceae* were positively correlated with serum parameters and hepatic inflammatory cytokines, and *Firmicutes*, *Verrucomicrobiota*, *Deferribacterota*, *Lachnospiraceae_ NK4A136_group*, *unclassified_Lachnospiraceae*, *Akkermansia*, and *Ligilactobacillus* were negatively correlated with these indexes. Specifically, *Bacteroidota* was significantly positively correlated with serum parameters and hepatic inflammatory cytokines (*p* < 0.05); *unclassified_Lachnospiraceae* was significantly negatively correlated with serum ALT, LPS, hepatic IL-1β, and hepatic IL-6 (*p* < 0.05). *Proteobacteria* was significantly positively correlated with serum TC (*p* < 0.05), while *Verrucomicrobiota*, *Deferribacterota*, *Lachnospiraceae_NK4A136_group*, and *Akkermansia* were significantly negatively correlated with serum TC (*p* < 0.05). *unclassified_Muribaculaceae* was significantly positively correlated with hepatic IL-1β (*p* < 0.05), while *Firmicutes* and *Ligilactobacillus* were significantly negatively correlated with hepatic IL-1β (*p* < 0.05).

## Discussion

This is the first study to find that folic acid could ameliorate alcohol-induced liver injury *via* GLA homeostasis to some extent. Folic acid ameliorated the destruction of the intestinal barrier and the dysbiosis of gut microbiota induced by alcohol, which subsequently reduced the leakage of LPS. Then, the activation of the TLR4 signaling pathway mediated by LPS in the liver was inhibited by folic acid, and consequently, liver injury induced by alcohol was improved.

ALD is a syndrome of liver injury associated with chronic intake of alcohol, which includes a range of alcohol-induced liver injury such as alcoholic fatty liver, alcoholic hepatitis, and alcoholic cirrhosis (26). The elevation of serum ALT and AST activity, and the infiltration of lipid droplets and inflammatory cells are early biochemical and pathological changes in ALD (27). In this study, the liver tissues of the MOD group showed obvious aggregation of lipid droplets and inflammatory cell infiltration through the histopathological examination, and liver index and serum ALT, AST, TG, and TC in the MOD group were significantly higher than those in the CON group, suggesting the alcohol-induced liver injury has occurred. Alcohol consumption is typically accompanied with folic acid deficiency (28). Our previous study has demonstrated that folic acid supplementation may relieve ethanol-induced Th17/Treg disbalance by altering Foxp3 promoter methylation patterns, which suggest that folic acid may be a feasible preventive strategy for ALD (29). In this study, in the FA group, histopathological liver changes induced by alcohol were improved, and liver index and the levels of serum ALT, AST, and TG were significantly lower than those in the MOD group. However, folic acid did not completely eliminate alcohol-induced liver injury compared with the CON group. The results showed that folic acid could improve alcohol-induced liver injury to some extent.

Notably, recent studies have demonstrated that modulated perturbations of the GLA emerged as a promising therapeutic option in the progression of ALD (30). In normal conditions, a balance between the intestinal barrier and pathogenic microorganisms in the gut lumen is maintained, which prevents harmful substances such as LPS translocation from the gut (31). In ALD, gut barrier disruption induced by alcohol results in a significant increase in gut permeability and gut leakiness, and subsequently, LPS is transported into the portal bloodstream and liver. LPS binds to endotoxin receptors to activate the liver TLR4 signaling pathway and consequently leads to the inflammatory injury. It is showed that phytochemicals such as semen hoveniae extract, ursolic acid, and astaxanthin could ameliorate alcohol-induced liver injury by affecting the GLA (32, 33). However, no study has focused on the ameliorating effect of folic acid on ALD by the mechanism of the GLA.

The destruction of the intestinal barrier facilitated the transfer of LPS from gut to the liver and general circulation (34). Alcohol consumption disrupts the intestinal barrier and increases gut permeability both in patients with ALD and in experimental models of ALD (34). Our results from the histopathological examination showed that the intestinal barrier was disrupted by alcohol, including the detachment of the intestinal epithelium and the destruction of intestinal villi integrity. In the FA group, the destruction of the intestine was ameliorated. Alcohol has been reported to increase intestinal epithelial permeability, mainly due to alcohol-induced alterations in the expression of tight junction proteins (35). This study showed the expression levels of tight junction proteins, including ZO-1, claudin 1, and occludin, were decreased in response to alcohol exposure. Importantly, alcohol-induced decreases in these indexes were notably raised by folic acid. Folic acid could alleviate alcohol-induced destruction of the intestinal barrier, which is beneficial to prevent harmful substances from entering the bloodstream.

The increase in intestinal permeability leads to the leakage of LPS, which was a recognized factor in the pathogenesis of ALD (36). In acute and chronic liver diseases, elevated serum LPS levels and the presence of a large number of inflammatory cytokines could be detected (37). Higher than normal levels of LPS can activate liver macrophages and extrahepatic macrophages to overproduce inflammatory cytokines (38), which could result in hepatocellular necrosis. Previous works showed that folic acid exerted anti-inflammatory activity in mice with NAFLD (39, 40). In this study, we confirmed the anti-inflammatory property of folic acid in alcohol-exposed mice. Folic acid not only decreased the accumulation of LPS in serum but also significantly inhibited the elevation of hepatic IL-1β, IL-6, and TNF-α levels induced by alcohol. Increasing evidence showed that intestinal permeation, translation of bacterial LPS, and activation of the TLR4-dependent signaling pathway in the liver are key mechanisms in the development of ALD (41, 42). The binding of LPS to the TLR4 receptor depends on MyD88 to trigger the initiation of a series of cascade reactions that activate interleukin receptor-associated kinase 1 (IRAK1) and its downstream tumor necrosis factor receptor-associated factor 6 (TRAF6) (43). In the cytoplasm, NF-κB remains inactive by forming a complex with NF-κB inhibitor protein (IκBα). With the activation of TRAF6, IκBα was phosphorylated that leading to the dissociation of NF-κB/IκBα complex and NF-κB activation, and finally specific target genes were activated and the expression of inflammatory cytokines were promoted (44). The results of this study showed that the expression levels of TLR4, MyD88, IRAK1, TRAF6, p-IκBα, and NF-κB proteins in the FA group were significantly decreased compared with the MOD group. Folic acid could reduce the intestinal leakage of LPS and inhibit the activation of the LPS/TLR4/NF-κB signaling pathway, which demonstrated that folic acid could exert anti-inflammatory effects to ameliorate alcohol-induced liver injury.

The dysbiosis of gut microbiota can trigger inflammation of ALD by compromising the intestinal barrier and increasing translocation of bacterial products LPS to the liver (45). The modulation of gut microbiota has potential to relieving liver diseases of different etiologies (28). Therefore, we conducted 16S rRNA gene sequencing to detect and analyze the changes of gut microbiota in each group of mice. We randomly selected five samples from each group to perform sequencing analysis by referring to the published animal studies (46, 47). Diversity analysis showed that there were differences of gut microbiota among the three groups, while the similarity of composition and structure of gut microbiota between the FA group and CON group was greater than those between the MOD group and CON group. At the phylum level, *Bacteroidota* and *Firmicutes* were the two most abundant phyla in gut microbiota, which is consistent with previous studies (32, 48). It was reported that following alcohol feeding, there was an overall decrease in *Firmicutes*, whereas the relative abundance of *Bacteroidota* increased in alcohol-fed mice (49). Acute-on-chronic alcohol administration induced shifts in various bacterial phyla in the mice, including a reduction in *Verrucomicrobiota* (50). Consistent with these studies, we found that alcohol exposure resulted in a significant increase in the relative abundance of *Bacteroidota*, and folic acid significantly increased the relative abundance of *Verrucomicrobiota*. Furthermore, folic acid significantly decreased the relative abundance of *Proteobacteria. Proteobacteria* is one of the harmful bacteria, the abundance of *Proteobacteria* in gut microbiota of patients with inflammatory bowel disease increased significantly, and patients with hepatic steatosis were reported to have a higher abundance of *Proteobacteria* (51, 52). At the genus level, the relative abundance of *Lachnospiraceae_NK4A136_group* and *Akkermansia* was significantly increased in the FA group compared with the MOD group, and the relative abundance of *unclassified_Lachnospiraceae* was significantly decreased in the MOD group compared with the CON group. *Lachnospiraceae_NK4A136_group* was related to a decline in intestinal injury (53). *Akkermansia* is often considered beneficial because it is associated with lower levels of inflammation, and it could improve intestinal barrier function (54). In addition, *Akkermansia* is involved in regulating the mucus produced by goblet cells, strengthening the intestinal barrier, and supporting the metabolic function of enterocytes; its depletion might exacerbate the toxic effects of alcohol and acetaldehyde on the intestinal barrier (55). *Lachnospiraceae* members are short-chain fatty acid propionate producers and microbiota composition modulators in the gut (56). And the increase of *unclassified_Lachnospiraceae* could be a signature of positive effects (57). To identify the specific bacterial taxa after folic acid supplementation, LEfSe analysis was conducted, with a threshold of 4.0 as the log LDA fraction of the distinguishing feature. The results showed that the relative abundance of *Prevotellaceae* and *Ileibacterium* was significantly higher in the MOD group than in other groups. Similar results have been observed in population studies, and a significant increase was found in the relative abundance of *Prevotellaceae* in microbiota of patients with alcohol-use disorders compared with healthy individuals (58). Another study pointed out that LPS biosynthesis may be associated with *Prevotellaceae* abundance (59). A previous studies on NAFLD showed an increased relative abundance of intestinal microbial pathogenic bacteria in mice, including *Ileibacterium*, *Turicibacter*, and *Faecalibaculum* (60). The relative abundance of *s_Bifidobacterium_animalis* was significantly higher in the FA group than in other groups. *Bifidobacterium* is a common probiotic that plays a vital role in the intestinal tract. Among a large number of bifidobacterial taxa, just a few, which include *Bifidobacterium_animalis*, have been exploited as health-promoting bacteria. In particular, *Bifidobacterium_animalis* strains have been extensively used as active ingredients in a variety of functional food species (61).

To further explore the potential relationship between gut microbiota and liver injury in alcohol-exposed mice, we performed correlation analysis between the relative abundance of gut microbiota and liver injury-related indexes. The results showed that *Bacteroidota*, *Proteobacteria*, and *uncultured_bacterium_f_Muribaculaceae* were significantly positively correlated with serum parameters and hepatic inflammatory cytokines, while *Firmicutes*, *Verrucomicrobia*, *Deferribacterota*, *Lachnospiraceae_NK4A136_group*, *uncultured_bacterium_f_Lachnospiraceae*, *Akkermansia*, and *Ligilactobacillus* were negatively correlation with them. Collectively, these results suggested that folic acid may ameliorate alcohol-induced liver injury by selectively promoting the relative abundance of specific beneficial microbiota and inhibiting the relative abundance of harmful microbiota. In this study, we did not perform gut microbiota sequencing and correlation analysis on all samples, which is a limitation for the study. The sequencing and correlation analysis may provide a high convincing result based on the larger sample size, and we will pay attention to this issue in our future studies.

## Conclusion

**FIGURE 9:**
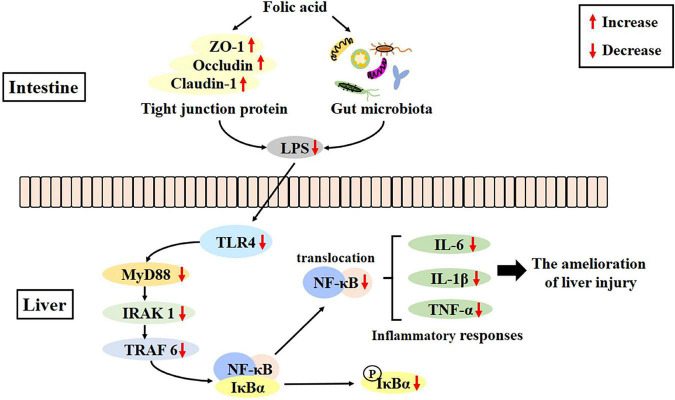
Folic acid ameliorates alcohol-induced liver injury *via* gut–liver axis (GLA) homeostasis, mainly including the improvement of the intestinal barrier, regulation of gut microbiota, and inhibition of liver inflammation.

Folic acid could regulate gut microecological dysbiosis, relieve intestinal barrier destruction, and inhibit the LPS-mediated activation of the TLR4/NF-κB signaling pathway, which, in turn, could ameliorate alcohol-induced liver injury to some extent (Figure 9). This is the first study demonstrating that the ameliorative effects of folic acid of alcohol-induced liver injury were probably associated with modulating the perturbations of the GLA in mice, which may serve as an excellent candidate for ALD prevention and uncover the underlying mechanisms involved.

## Data availability statement

The data presented in this study are deposited in the NCBI repository, accession number: PRJNA874577.

## Ethics statement

The animal study was reviewed and approved by Qingdao University Laboratory Animal Welfare Ethics Committee.

## Author contributions

HQZ and YZ participated in data collection, performed the analyses, and wrote the manuscript. HCZ and HZ conducted animal experiments. YW, XZ, and JZ performed in data analysis. PW, LS, and HZZ performed microbiota data pre-processing. HL participated in the study design and was responsible for overall study coordination. All authors contributed to the article and approved the submitted version.

## Footnotes

1
https://www.ncbi.nlm.nih.gov/sra/PRJNA874577
